# Phthalate exposure promotes chemotherapeutic drug resistance in colon cancer cells

**DOI:** 10.18632/oncotarget.23481

**Published:** 2017-12-08

**Authors:** Hsin-Pao Chen, Yung-Kuo Lee, Shih Yin Huang, Pei-Chun Shi, Ping-Chi Hsu, Chuan-Fa Chang

**Affiliations:** ^1^ Institute of Basic Medical Science, College of Medicine, National Cheng Kung University, Tainan 70101, Taiwan; ^2^ Department of Surgery, E-DA Hospital, I-Shou University, Kaohsiung 824, Taiwan; ^3^ Department of Medical Laboratory Science and Biotechnology, College of Medicine, National Cheng Kung University, Tainan 70101, Taiwan; ^4^ Center of Infectious Disease and Signaling Research, College of Medicine, National Cheng Kung University, Tainan 70101, Taiwan; ^5^ Department of Safety, Health and Environmental Engineering, National Kaohsiung First University of Science and Technology, Kaohsiung 811, Taiwan

**Keywords:** colon cancer, DEHP, MEHP, drug resistance, phthalates

## Abstract

Phthalates are widely used as plasticizers. Humans can be exposed to phthalates through ingestion, inhalation, or treatments that release di(2-ethylhexyl) phthalate (DEHP) and its metabolite, mono(2-ehylhexyl) phthalate (MEHP), into the body from polyvinyl chloride-based medical devices. Phthalate exposure may induce reproductive toxicity, liver damage, and carcinogenesis in humans. This study found that colon cancer cells exposed to DEHP or MEHP exhibited increased cell viability and increased levels of P-glycoprotein, CD133, Bcl-2, Akt, ERK, GSK3β, and β-catenin when treated with oxaliplatin or irinotecan, as compared to control. The P-glycoprotein inhibitor, tariquidar, which blocks drug efflux, reduced the viability of DEHP- or MEHP-treated, anti-cancer drug-challenged cells. DEHP or MEHP treatment also induced colon cancer cell migration and epithelial-mesenchymal transformation. Elevated stemness-related protein levels (β-catenin, Oct4, Sox2, and Nanog) and increased cell sphere sizes indicated that DEHP- or MEHP-treated cells were capable of self-renewal. We also found that serum DEHP concentrations were positively correlated with cancer recurrence. These results suggest phthalate exposure enhances colon cancer cell metastasis and chemotherapeutic drug resistance by increasing cancer cell stemness, and that P-glycoprotein inhibitors might improve outcomes for advanced or drug-resistant colon cancer patients.

## INTRODUCTION

Colon cancer is the third most common cancer in the world and the second leading cause of cancer-related death in the western world [1]. In Taiwan, colon cancer incidence has increased dramatically over the last two decades, and causes more than 4,000 deaths annually [2]. Current colon cancer treatment options include surgery, radiation therapy, and chemotherapy. However, chemotherapeutic resistance, cancer recurrence, and metastasis reduce the five-year survival rate in patients with late-stage disease [3–5]. Approximately 20% of metastatic colon cancer patients experience disease recurrence, typically involving the liver or lung [6]. Various factors may promote cancer recurrence, including obesity, pre-operative conditions, number of positive lymph nodes, certain tumor markers and genetic factors, and the presence of drug-resistant cancer stem/stem-like cells (CSCs) [7]. Resistance to chemotherapeutic agents is a major problem in cancer treatment. Multi-drug resistance (MDR), which may occur during initial chemotherapeutic treatment or during disease recurrence, is controlled in part by a group of ATP-binding cassette (ABC) transporters involved in drug uptake and efflux [8]. Cancer cell drug resistance mechanisms can include increased drug efflux, reduction of drug uptake, growth signaling activation, and inhibition of apoptosis signaling via induction of anti-apoptotic molecules [9, 10].

Preparation and administration of intravenous anticancer drugs, parenteral nutrition, and other medical treatments in cancer patients are commonly performed using polyvinyl chloride (PVC) plastic bags and tubing sets containing di(ethylhexyl) phthalate (DEHP) [11, 12], a manufactured chemical commonly added to plastics. DEHP is a ubiquitous environmental contaminant to which humans are exposed through multiple routes [13, 14], and is released from plastics into the environment via direct release, migration, evaporation, leaching, and abrasion [15]. DEHP reportedly acts as a steroid and xenobiotic receptor (SXR) ligand, activating multidrug resistance 1 (MDR1) gene transcription in the human colon adenocarcinoma-derived cell line, LS174T [16]. High DEHP concentrations can also induce resistance in sarcoma cells by decreasing anticancer drug cytotoxicity and increasing MDR expression [17]. Thus, DEHP leaching from PVC medical devices may induce drug resistance in certain cancer cells, although the clinical impact of DEHP exposure via blood and blood components transfusion remains poorly understood [16].

Several groups have reported the effects of phthalates on reproductive toxicity, liver damage, and carcinogenesis, but phthalate mechanisms of action in tumorigenesis, tumor progression, and drug resistance are still unclear [18–26]. This study evaluated serum DEHP/MEHP levels in colon cancer patients, and found that DEHP/MEHP concentrations increased with cancer stage. Additionally, DEHP/MEHP treatment not only enhanced colon cancer cell viability and migration *in vitro*, but also induced epithelial-mesenchymal transformation (EMT) and expression of drug resistance-associated proteins. DEHP/MEHP-promoted drug resistance was ameliorated by treating cells with an efflux pump inhibitor. We also assessed stemness-related protein expression (β-catenin, Oct4, Sox2, and Nanog) and self-renewal in DEHP/MEHP-treated colon cancer cells. Our findings suggest that phthalate exposure promotes colon cancer cell drug resistance and tumor metastasis by increasing cancer stemness.

## RESULTS

### DEHP and MEHP induced drug resistance in HCT116 and SW480 cells

**Figure 1:**
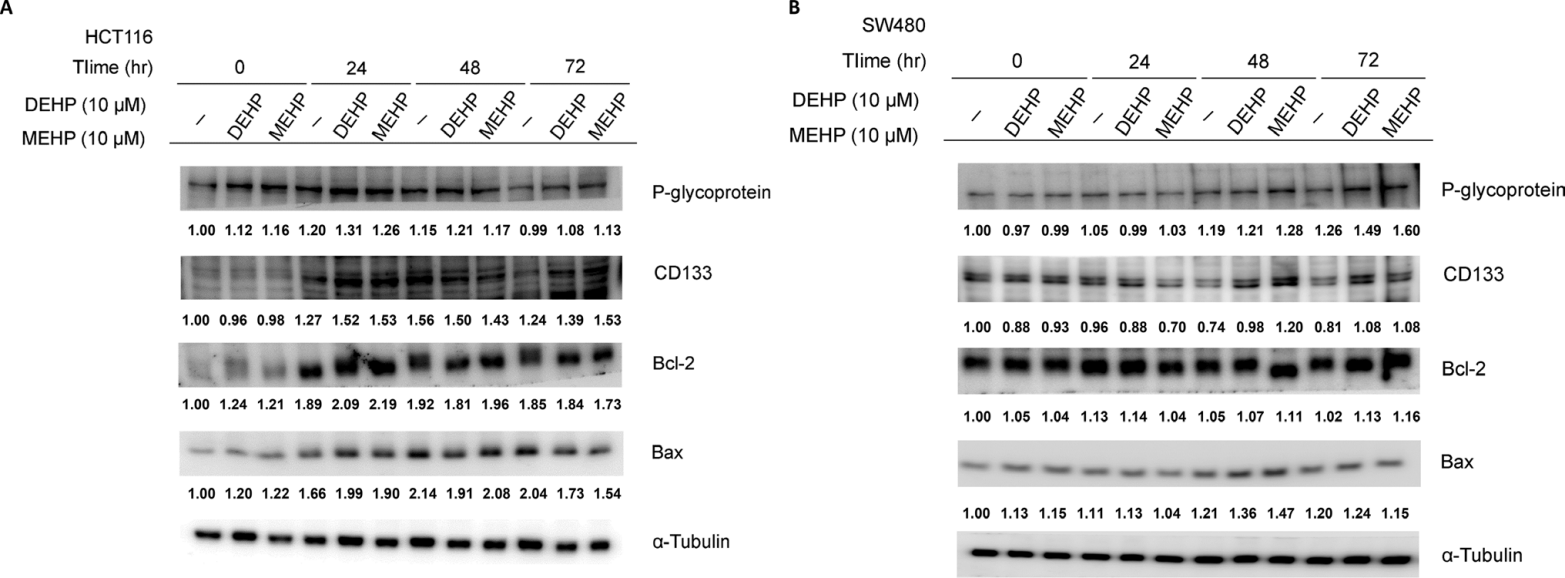
Phthalate treatment upregulated drug resistance- and anti-apoptosis-related proteins in HCT116 and SW480 cells Cells were incubated with or without DEHP or MEHP (10 μM) for 0, 24, 48, or 72 h. Western blotting results showed that P-glycoprotein, CD133, and Bcl-2 levels, and the Bcl-2/Bax ratio were increased in treated cells. (−) indicates cells grown without DEHP/MEHP treatment.

Drug resistance-associated protein levels were evaluated in DEHP or MEHP-treated HCT116 and SW480 colon cancer cells. While P-glycoprotein levels increased from 0 to 72 h in untreated, DEHP- or MEHP-treated SW480 cells, the levels of P-glycoprotein increased from 0 to 24 h and declined to basal level after 72 h in untreated, DEHP- or MEHP-treated HCT116 cells (Figure 1A–1B). Besides, P-glycoprotein levels of DEHP- or MEHP-treated cells were higher than untreated cells at most of the time points. The expression of CD133 was elevated after 24 h and maintained at high level to 72 h in DEHP- or MEHP-treated HCT116 cells (Figure 1A). However, levels of CD133 increased from 0 to 48 h and dropped after 72 h in untreated HCT116 cells (Figure 1A). In DEHP- or MEHP-treated SW480 cells, CD133 levels decreased slightly and then increased at 48/72 h (Figure 1B). Unlike HCT116 cells, the expression of CD133 in untreated SW480 cells decreased from 0 to 72 h (Figure 1B). In addition, the expression patterns of Bcl-2 and Bax were similar in untreated, DEHP- or MEHP-treated cells. The levels of P-glycoprotein and CD133 in DEHP- or MEHP-treated cells were higher than untreated cells after 72 h.

**Figure 2:**
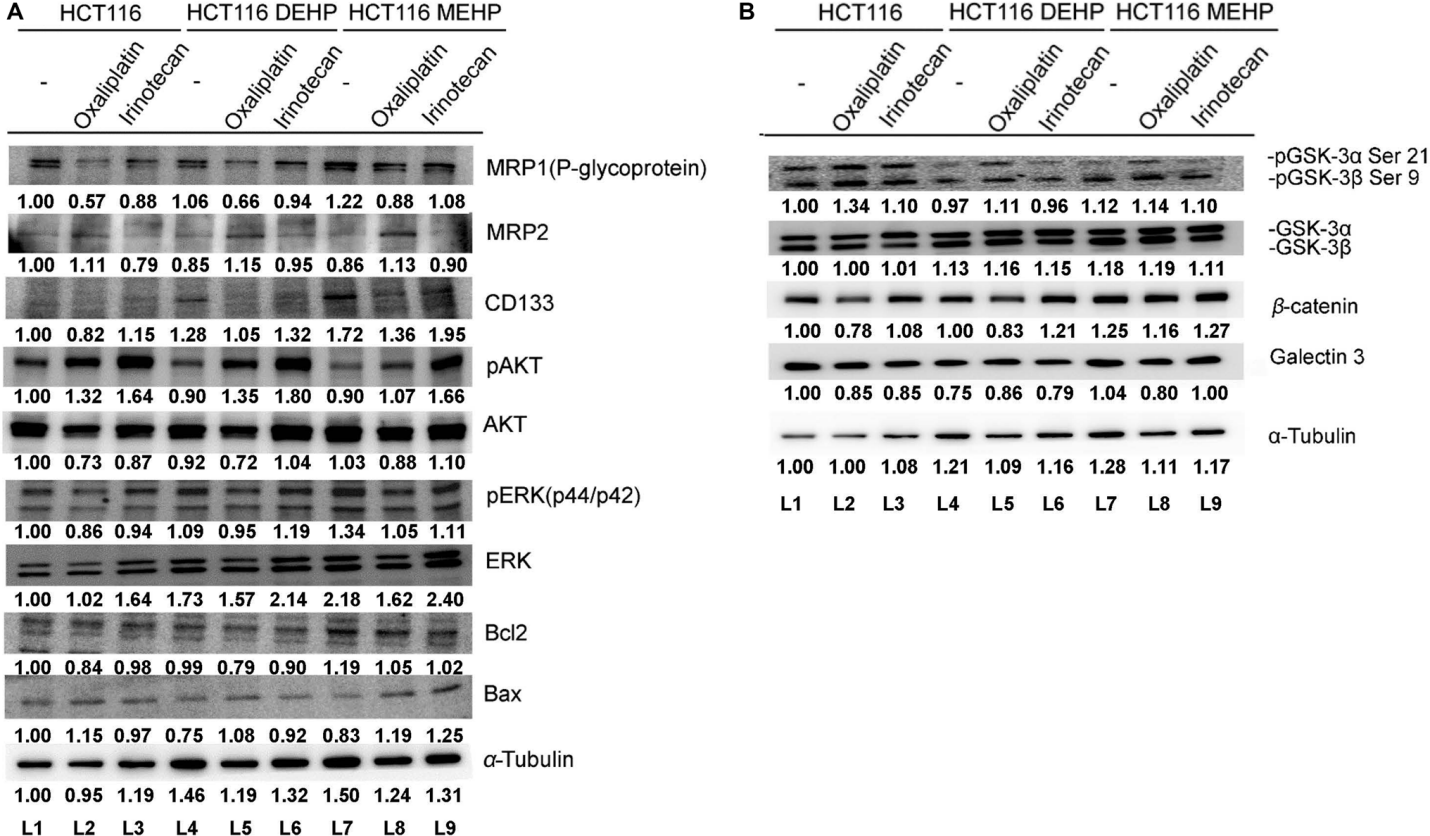
Oxaliplatin or irinotecan treatment enhanced DEHP/MEHP-induced drug resistance in HCT116 cells Untreated and DEHP- or MEHP-treated HCT116 cells were cultured with or without anticancer drugs (oxaliplatin or irinotecan, 10 μM). After 48 h, drug resistance-, proliferation-, and apoptosis-associated proteins were analyzed via western blotting. P-glycoprotein, CD133, and ERK levels (**A**) as well as GSK3β and β-catenin levels (**B**) were increased in anticancer drug-challenged, DEHP/MEHP-treated cells. (−) indicates cells grown for 6 months without DEHP/MEHP treatment. Numbers indicate densitometric analysis of protein expression levels normalized to corresponding control levels (L1) and α-tubulin (the last row).

HCT116 cells were then treated with DEHP or MEHP for > 6 months to investigate the effects of long-term exposure. Consistent with short-term treatment results, P-glycoprotein, CD133, and ERK levels were higher in DEHP- or MEHP-treated cells. However, the level of multidrug resistance protein 2 (MRP2), were decreased in a long-term treatment. In addition, pERK levels and the Bcl-2/Bax ratio were elevated in MEHP-treated cells (L1 and L7; Figure 2A). pAkt, Akt, Bax, pGSK3β, GSK3β, β-catenin and galectin-3 levels were all reduced in DEHP- or MEHP-treated cells (L1, L4, and L7; Figure 2A–2B). We then challenged phthalate-treated cells with oxaliplatin or irinotecan, two FDA-approved anti-colon cancer drugs. Irinotecan IC_50_ values for untreated, DEHP-, or MEHP-treated HCT116 cells were 80, 98, and 106 mM, respectively (Supplementary Figure 1A). Irinotecan IC_50_ values for untreated, DEHP-, or MEHP-treated SW480 cells were 56, 68, and 102 mM, respectively (Supplementary Figure 1B). Data are reported as percentage of cell viability compared to controls (100%). We found that oxaliplatin treatment decreased the expression of P-glycoprotein, Akt, pERK, Bcl-2, Bax, and Bcl-2/Bax ratio, and increased the expression of MRP2, CD133, pAkt, ERK, and Bax in phthalate untreated cells (L1 and L2; Figure 2A). Irinotecan treatment decreased the expression of P-glycoprotein, MRP2, CD133, Akt, pERK, Bcl-2, and Bax but increased pAkt, ERK and Bcl-2/Bax ratio in phthalate untreated cells (L1 and L3; Figure 2A).

Western blotting results showed that P-glycoprotein and CD133 levels were increased in DEHP- or MEHP-treated, oxaliplatin- or irinotecan-challenged HCT116 cells compared with oxaliplatin- or irinotecan-challenged control cells (L2, L5, and L8/L3, L6, and L9; Figure 2A). Cell proliferation-related proteins, including Akt, pERK, ERK (L2, L5, and L8/L3, L6, and L9; Figure 2A), GSK3β, and β-catenin (L2, L5, and L8/L3, L6, and L9; Figure 2B), were also upregulated, while phospho-GSK3β and galectin-3 were downregulated compared with oxaliplatin- or irinotecan-challenged control cells (L2, L5, and L8/L3, L6, and L9; Figure 2B). An increased Bcl-2/Bax ratio indicated enhanced cell survival in DEHP-treated, oxaliplatin- or irinotecan-challenged cells and MEHP-treated, oxaliplatin-challenged cells (L2, L5, and L8/L3 and L6; Figure 2A). These findings suggest that phthalate treatment promoted drug resistance in colon cancer cells via drug efflux machinery.

### Tariquidar reduced phthalate-treated HCT116 and SW480 cell viability

**Figure 3:**
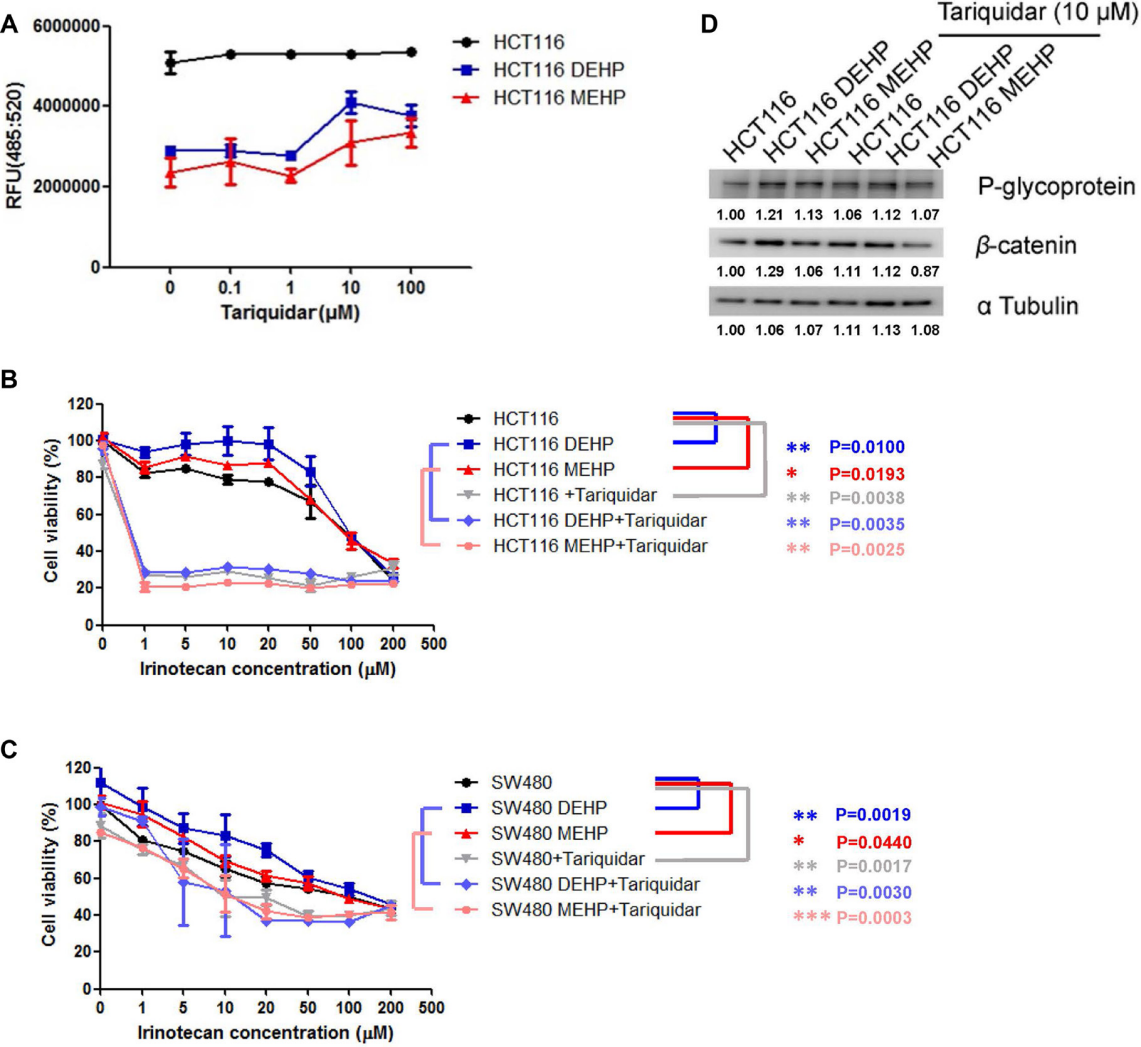
Tariquidar treatment reduced DEHP/MEHP-induced drug resistance by blocking drug efflux in HCT116 and SW480 cells Untreated and DEHP- or MEHP-treated HCT116 cells were incubated with tariquidar (0, 0.1, 1, 10 or 100 μM) for 24 h. Fluorescent dye was then added and incubated for 1 h. Tariquidar treatment increased intracellular fluorescent dye accumulation (**A**) DEHP- or MEHP-treated HCT116 and SW480 cells were incubated with or without tariquidar (10 μM) for 24 h and then challenged with irinotecan (0, 1, 5, 10, 20, 50, 100, or 200 μM) for 48 h. DEHP/MEHP treatment enhanced cell viability, and tariquidar pretreatment decreased viability in anti-cancer drug-challenged HCT116 (**B**) and SW480 cells (**C**) P-glycoprotein was not affected but β-catenin was downregulated following tariquidar treatment (**D**) Data are presented as means ± standard deviation (SD) from at least three independent experiments. Numbers indicate densitometric analysis of protein expression levels normalized to corresponding control levels (L1) and α-tubulin (the last row).

To assess whether drug efflux machinery contributed to DEHP- or MEHP-induced drug resistance, phthalate-treated cells were incubated with tariquidar (0, 0.1, 1, 10 or 100 μM), a P-glycoprotein inhibitor with low toxicity in control HCT116 and SW480 cells (Supplementary Figure 2A–2B) [27]. DEHP- or MEHP-treated cells effluxed more fluorescence dye than untreated cells, but fluorescent dye deposited inside cells increased with increasing tariquidar concentrations (Figure 3A). Tariquidar (10 μM) was then incubated with untreated, DEHP-, or MEHP-treated cells challenged with irinotecan (1, 5, 10, 20, 50, 100 or 200 μM), and cell viability was measured via MTT assay. While DEHP or MEHP treatment increased cancer cell viability by 5–20% in 1–20 μM irinotecan-treated cells (blue square and red triangle) compared with control cells (black circle), tariquidar reduced viability at all irinotecan concentrations (Figure 3B–3C). DEHP or MEHP treatment induced cancer cell viability could also be observed at low dose of tariquidar treatment (Supplementary Figure 3A–3B). P-glycoprotein expression was not affected by tariquidar in DEHP- or MEHP-treated colon cancer cells (Figure 3D and Supplementary Figure 3C).

### Phthalate exposure promoted HCT116 cell migration via EMT

**Figure 4:**
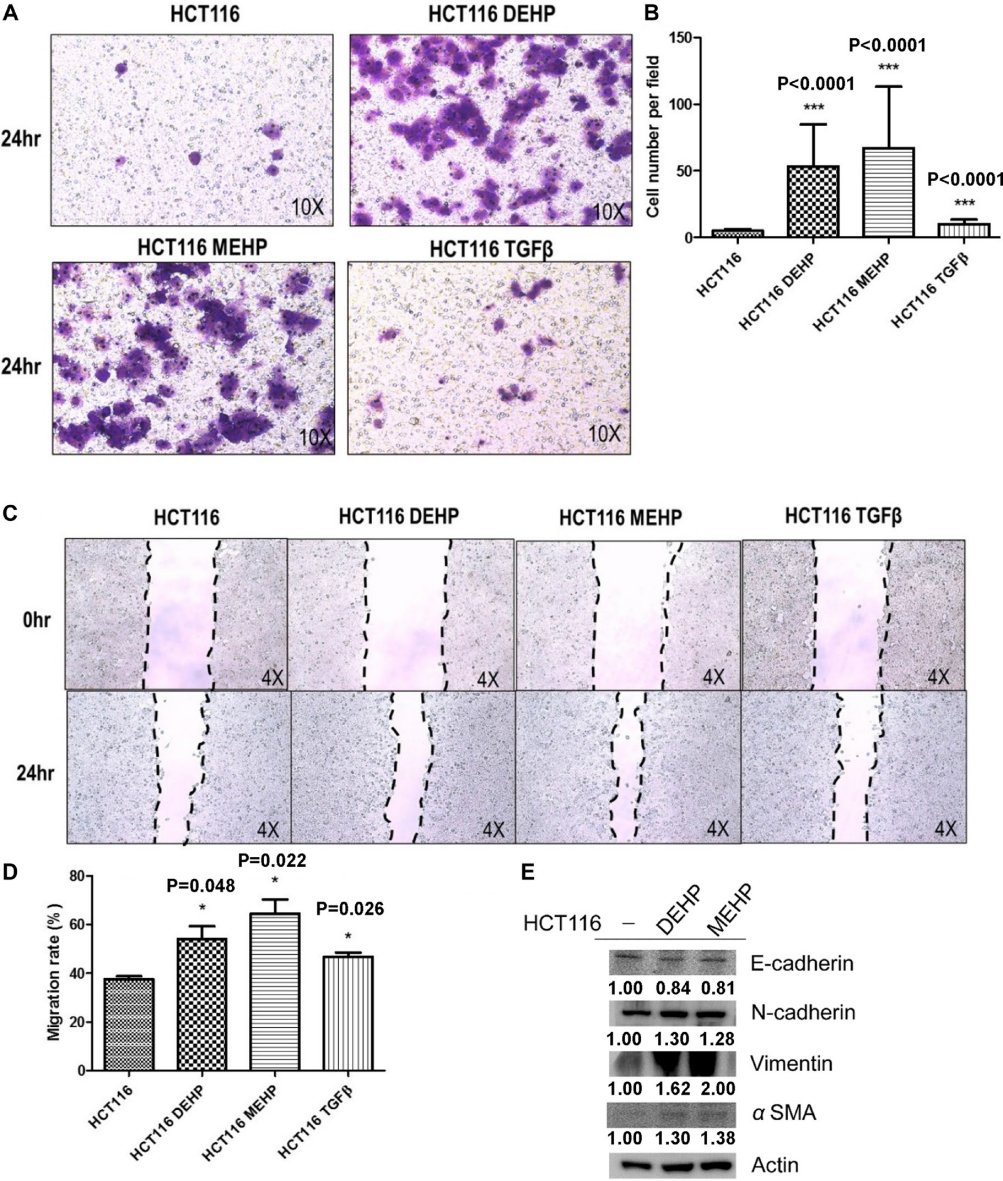
DEHP/MEHP treatment induced EMT and enhanced HCT116 cell migration Untreated and DEHP- or MEHP-treated HCT116 cells were seeded into transwell upper chambers, and 500 µl medium containing 10% FBS was added into lower chambers. TGFβ (10 mM) treatment was used as positive control. After 24 h, migrated cells were counted. DEHP-, MEHP-, or TGFβ-treated cells migrated more than untreated controls (**A**, **B**) Wound-healing assay results showed that DEHP-, MEHP-, or TGFβ-treated HCT116 cells migrated more than untreated controls (**C**, **D**) EMT markers were analyzed via western blotting (**E**) DEHP, MEHP, or TGFβ treatment downregulated E-cadherin and upregulated N-cadherin, vimentin, and α-smooth actin (α-SMA). Data are presented as means ± standard deviation (SD) from at least three independent experiments. ^***^P < 0.001, ^#^*P* < 0.05 compared with HCT116.

DEHP- or MEHP-treated or untreated cells were seeded on transwell upper chambers and incubated for 24 h. TGFβ treatment, which promotes tumor cell migration, was used as positive control. Cells that migrated from upper chambers into lower chambers were fixed, stained, and counted. The numbers of DEHP- or MEHP-treated cells on the lower chambers were higher than untreated and positive control cells (Figure 4A–4B). DEHP- or MEHP-treated cells also reduce the wound-healing assay gap area more than untreated and positive control cells (Figure 4C–4D). To determine whether DEHP or MEHP promoted cell migration through EMT, EMT markers were analyzed via western blotting. E-cadherin downregulation and N-cadherin, vimentin, and α-smooth actin (α-SMA) upregulation together indicated that phthalate-treated colon cancer cells underwent EMT (Figure 4E).

### DEHP/MEHP exposure promoted HCT116 cell sphere formation and upregulated stemness-related proteins

**Figure 5:**
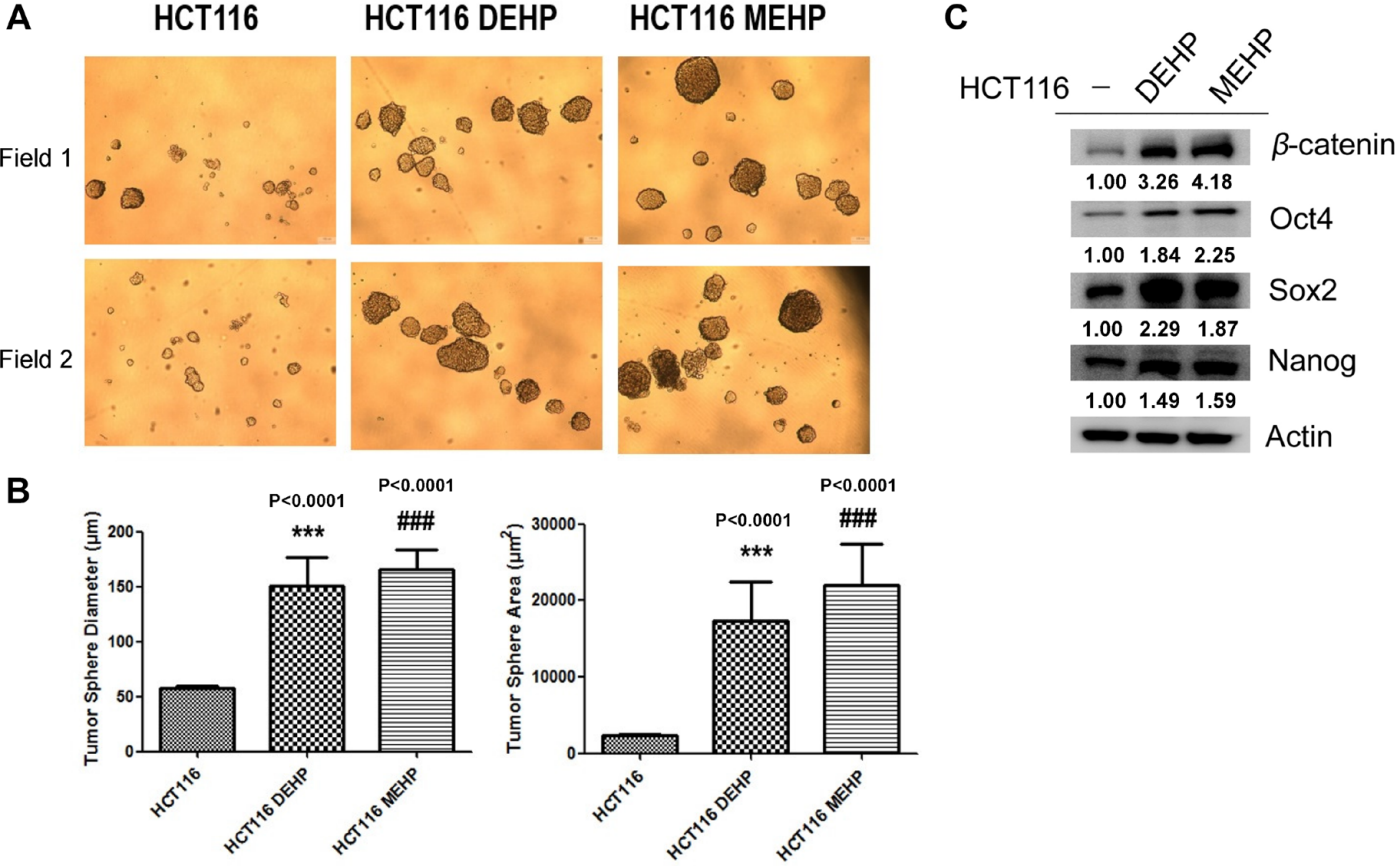
DEHP/MEHP treatment increased sphere formation and upregulated stemness-related proteins in HCT116 cells Sphere-forming capability was measured in untreated and DEHP- or MEHP-treated HCT116 cells (**A**) DEHP or MEHP treatment increased sphere diameters and areas (**B**) and upregulated stemness-related proteins (β-catenin, Oct4, Sox2, and Nanog) (**C**) Data are presented as means ± standard deviation (SD) from at least three independent experiments. ^***^P < 0.001, ^###^*P* < 0.001 compared with HCT116.

We analyzed cancer stemness-related protein expression and self-renewal in DEHP- or MEHP-treated HCT116 and SW480 cells. Diluted cell (1 × 10^3^ cells) suspensions were seeded in 96-well plates, and sphere sizes and surface areas were measured and calculated after 2 weeks. DEHP or MEHP treatment not only increased sphere radius and area, but also induced expression of stemness-related proteins, including β-catenin, Oct4, Sox2, and Nanog, in both HCT116 (Figure 5A–5C) and SW480 cells (Supplementary Figure 4A–4B). Our findings suggest that DEHP or MEHP exposure enhances colon cancer cell drug resistance and migration by promoting stemness.

### Serum DEHP/MEHP concentrations were elevated in colon cancer patients

**Figure 6:**
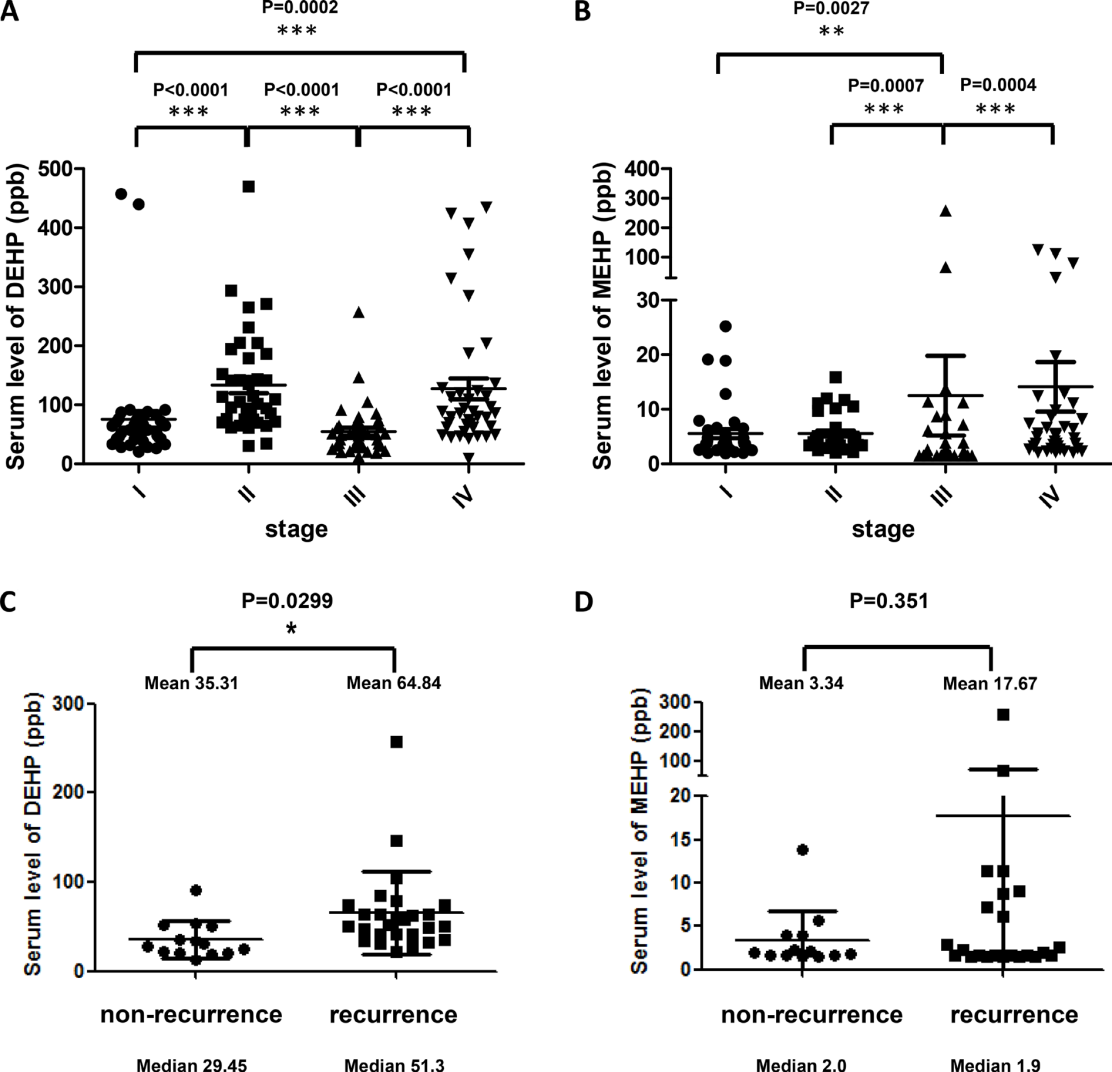
Serum phthalate levels in colon cancer patients Scatter dot plots shows the distribution of serum DEHP (**A**) and MEHP (**B**) levels in colon cancer patients before first treatment. Mean serum DEHP concentrations from stages I to IV were 75.5, 113.4, 54.8, and 127 ppb, respectively. Median serum DEHP concentrations from stage I to IV were 53.3, 107.3, 48.5, and 85.9 ppb, respectively. Mean serum MEHP concentrations from stages I to IV were 5.6, 5.6, 12.5, and 14.1 ppb, respectively. Median serum MEHP concentrations from stage I to IV were 3.9, 4.8, 5.2, and 5.3 ppb, respectively. Scatter dot plots showed the distribution of serum DEHP (**C**) and MEHP (**D**) levels from non-recurrent and recurrent colon cancer patients. Serum DEHP concentration were significantly higher in recurrent cancer patients. ^***^P < 0.001, ^**^P < 0.01, ^*^P < 0.05 (Mann-Whitney *t*-test).

We collected 160 patient serum samples (40 per stage, from I to IV) from the Bio-Bank of the Medical Research Department, E-DA Hospital. Serum DEHP/MEHP levels were measured via LC-MS. Mean serum DEHP concentrations were higher in stage II and IV (113.4 and 127 ppb, respectively) than stage I and III patients (75.5 and 54.8 ppb, respectively; Figure 6A). Mean serum MEHP concentrations increased with cancer stage (stages I to IV: 5.6, 5.6, 12.5, and 14.1 ppb, respectively; Figure 6B). Median serum DEHP concentrations from stage I to IV were 53.3, 107.3, 48.5, and 85.9 ppb, respectively, and median serum MEHP concentrations from stage I to IV were 3.9, 4.8, 5.2, and 5.3 ppb, respectively. Although serum phthalates seem increased accompanied with cancer stages, the distribution of the DEHP and MEHP levels were similar among the four cancer stages except some outlier patients. To answer whether phthalate exposure impinge on colon cancer drug resistance, we further divided patients into two groups, non-recurrence and recurrence. We found that serum DEHP concentrations were significantly higher in patients with cancer recurrence (Figure 6C), and serum MEHP levels had no correlation with cancer recurrence except maybe for a small number of outliers (Figure 6D).

## DISCUSSION

Phthalate exposure can negatively affect human health. Some phthalates are developmental and reproductive toxins that modulate endogenous fetal testicular testosterone production [28]. Studies in mice suggest that others, such as DEHP or MEHP, are potential human carcinogens [18–20, 26]. DEHP can be found in building and construction materials, clothing, furnishings, toys, and food or medical contact materials. Human exposure to DEHP can occur via skin contact, inhalation, ingestion, and intravenous routes [13, 29, 30]. DEHP/MEHP serum or urine levels can be high when DEHP is released from medical equipment to neonatal intensive care unit patients [30, 31]. The estimated average total daily exposure to DEHP for adult is 0.21–2.1 mg/day (in a 70-kg adult) [14, 32], and patient serum DEHP is increased to as much as 1,200 ppb after transfusion [33]. DEHP and MEHP can also pass through the placenta and shorten the gestational period of a developing fetus [23]. DEHP exposure may induce inflammation and proto-oncogene upregulation, resulting in tumorigenesis via increased oxidative stress [21]. DEHP and MEHP can also promote tumor cell migration and invasion by activating MMP2 [34]. Because plastics are widely used in food packaging in Taiwan, DEHP exposure is often higher in the Taiwanese population than in others [35].

P-glycoprotein and MRPs increase tumor cell survival and delay apoptosis [36, 37]. Upregulation of P-glycoprotein, ABC transporters, and MRPs, such as MRP1 and MRP2, are the main causes of pump-related MDR. P-glycoprotein upregulation is positively correlated with β-catenin, c-myc, and cyclin D1 expression, and may enhance *MDR1* expression through TCF4/β-catenin responsive elements found in the *MDR1* promoter [38–40]. Although other groups found that low dose phthalate exposure induces colon and breast cancer cell proliferation [41, 42], neither DEHP nor MEHP (3–100 μM) affected cell proliferation in our study (Supplementary Figure 5). Besides, the very similar P-glycoprotein, CD133, Bcl-2 and Bax expression trends in short-term phthalate treated cells may be the result of cell starvation (Figure 1A and 1B).

CD133 is a common hematopoietic stem cell marker which has been widely used as a marker to identify and isolate cancer initiating cells including brain, breast, prostate and colorectal cancer, especially the metastatic colon cancer [43–48]. The biological significance of CD133 is still unclear. Park et al. reported that CD133 expression is negatively regulated by p53, and suppression of CD133 expression may suppress p53 activity. In addition, CD133 depletion also suppresses tumor cell proliferation, colony formation, and the expression of stemness transcription factors including NANOG, OCT4, SOX2, and c-MYC [49]. Wei et al. also indicated that phosphorylated CD133 could interact with p85 and activate the PI3K/Akt signal pathway, which results in the increased tumorigenic capacity and stemness genes in glioma stem cells [50]. We found that DEHP/MEHP exposure induced resistance to oxaliplatin and irinotecan in HCT116 and SW480 colon cancer cells. Increased P-glycoprotein and CD133 expression in phthalates treated HCT116 and SW480 cells after 72 h suggested the induction of drug resistance and stemness/self-renewal abilities. In long-term phthalate treatment, P-glycoprotein expression was downregulated without anti-cancer drug treatment and upregulated after anti-cancer drug challenge. Combined with tariquidar treatment results in Figure 3, phthalate-treated cells overexpressed P-glycoprotein and pumped out fluorescent MDR indicator dye and irinotecan at higher levels than controls suggested that DEHP/MEHP exposure enhances drug resistance in part through ABC transporter upregulation. On the other hand, the levels CD133 was elevated in short- and long-term phthalates treated HCT116 and SW480 cells (after 72 h and more than 6 months, Figures 1A and 2A). DEHP/MEHP exposure also induced higher CD133 expression in anti-cancer drug challenged cells than control cells (Figure 2A). Increased CD133 and stemness-associated protein levels (β-catenin, Oct4, Sox2, and Nanog) and enhanced sphere size/formation (self-renewal) suggest that DEHP/MEHP treatment promotes colon cancer cell stemness (Figure 5A-5C) [51, 52]. Although we previously demonstrated that galectin-3 overexpression upregulates upstream efflux pump protein and downregulates apoptosis signaling in anti-cancer drug-stimulated Caco2 cells [10], galectin-3 downregulation in phthalate-treated, anti-cancer drug-challenged HCT116 cells suggests a unique regulatory pathway for phthalate-induced drug resistance.

Oxaliplatin is a platinum derivative and standard chemotherapeutic used to treat many digestive cancers, especially colon cancer [53–55]. Oxaliplatin induces DNA lesions, inhibits DNA and RNA synthesis, and triggers immunologic reactions that lead to cell apoptosis [56]. Irinotecan, which targets topoisomerase I, is also a potent antitumor drug used against a wide range of cancers [57–59]. However, tumor resistance to both drugs has been reported in advanced cancer patients [60–63]. Multidrug resistance protein overexpression is one mechanism by which tumor cells resist oxaliplatin and irinotecan treatment [61, 64, 65]. We found that DEHP/MEHP-induced P-glycoprotein overexpression promotes anti-cancer drug resistance in oxaliplatin or irinotecan challenged cells. The addition of tariquidar also significantly reduced DEHP/MEHP-induced cell viability. Taken together, our results suggest that the use of a P-glycoprotein inhibitor combined with oxaliplatin or irinotecan chemotherapy should be an alternative therapy for advanced or drug resistant colon cancer cases [66].

The relationship between phthalates and cell migration has not been well studied. Hsieh, *et al.* found that phthalates induce proliferation and invasion in estrogen receptor-negative breast cancers and stimulate EMT in human breast epithelial stem cells [24, 67]. Phthalates can also promote invasion and metastasis in human neuroblastoma cells [68], and long term phthalate treatment induced metastasis and increased the proportion of cancer stem-like cells in an animal model [69, 70]. According to our findings, DEHP- or MEHP-treated colon cancer cells not only exhibited drug resistance, but also enhanced migration and EMT.

Finally, we found that the highest serum DEHP/MEHP concentrations were observed in stage IV colon cancer patients, and serum DEHP concentrations were positively correlated with cancer recurrence. Although the serum phthalates did not show correlation with cancer stages because of some outlier patients in current study, large-scale clinical studies can be undertaken to improve and validate our findings in future.

**Figure 7:**
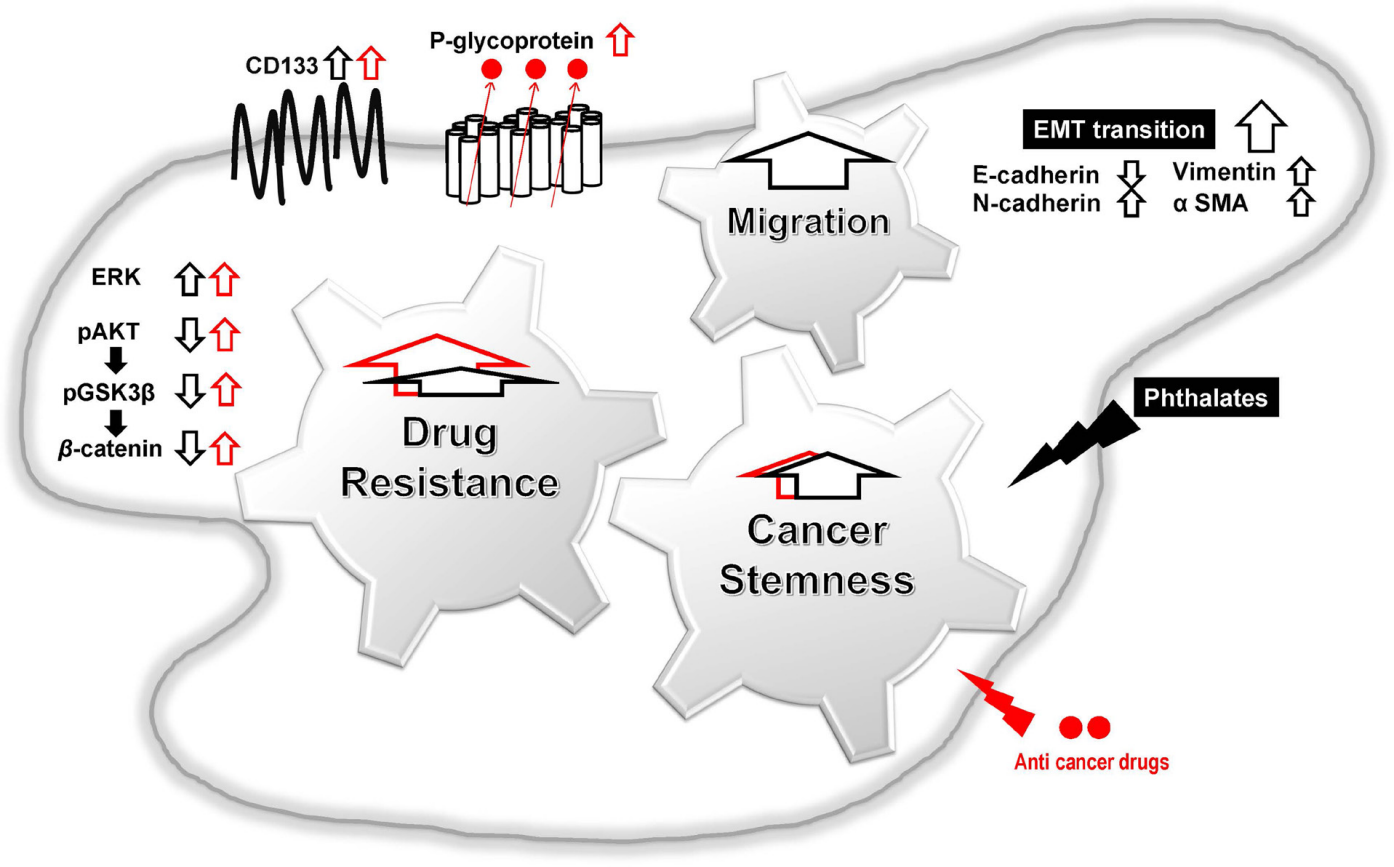
Proposed mechanisms of phthalate-promoted drug resistance and cell migration in colon cancer Colon cancer cell exposure to DEHP or MEHP upregulates stemness-, drug resistance-, and EMT-related proteins. Induced proteins successfully pump out anti-cancer chemotherapeutics, reduce apoptosis, and enhance cell survival in DEHP/MEHP-exposed cancer cells. DEHP/MEHP exposure also promotes cancer cell migration.

In conclusion, this is the first report demonstrating that DEHP or MEHP treatment promotes stemness, upregulates drug resistance-related proteins, and enhances migration and EMT in colon cancer cells (Figure 7). Our results suggest that the use of DEHP-containing medical devices should be regulated to reduce patient DEHP exposure, and that P-glycoprotein inhibitors might improve outcomes for advanced or drug-resistant colon cancer patients.

## MATERIALS AND METHODS

### Cell culture and experimental conditions

HCT116 colon cancer cells (purchased from the Food Industry Research and Development Institute) were maintained in McCoy’s 5A medium (Gibco Life Technologies, USA) supplemented with 100 unit/mL penicillin, 100 μg/mL streptomycin, and 10% fetal bovine serum (FBS; Hyclone, USA) at 37°C and 5% CO_2_. SW480 colon cancer cells (purchased from the Food Industry Research and Development Institute) were maintained in Leibovitz’s L-15 medium (Gibco Life Technologies) supplemented with 100 unit/mL penicillin, 100 μg/mL streptomycin, and 10% FBS (Hyclone) at 37°C and 5% CO_2_. Cultured cells were passaged every three days. Phthalates (10 μM DEHP or MEHP) [16, 22] or vehicle was added to fresh culture medium at each passage over the course of > 6 months.

### Serum DEHP/MEHP concentrations

DEHP/MEHP concentrations were measured in serum samples form the Bio-Bank of the Medical Research Department, E-DA Hospital. Before analysis, serum samples were thawed at 4°C for 24 h (Supplementary Table 2). Serum (100 μL) was added to 200 μL of MeOH containing 75 ng/mL of DEHP-d_4_ and ^13^C_4_-MEHP, placed in a 1.5-mL microcentrifuge tube, and vortexed vigorously for 5 min to precipitate proteins. The mixture was then centrifuged at 15,000 × g at 4°C for 10 min, and the supernatant was transferred to a 500 μL sample vial for DEHP and MEHP analysis.

We used a Waters UPLC-MS/MS system to separate and detect DEHP and MEHP. An ACQUITY UPLC Isolator (2.1 × 50 mm) was applied for elimination of background phthalates, and an ACQUITY UPLC^®^ BEH C18 column (2.1 mm × 50 mm, 1.7 µm) was used for separation. Five μL of prepared sample were injected for analysis at a flow-rate of 0.5 mL/min. The mobile phases were MeOH (mobile phase A) and 5 mM of NH4Ac in Milli-Q water (mobile phase B). The initial condition (0 min) was 20% mobile phase A and 80% mobile phase B, with a gradient from 0.5–2.5 min bringing mobile phase A to 90% and mobile phase B to 10%. The column and sample tray temperatures were 45°C and 4°C, respectively.

### Western blotting

HCT116 cells were cultured in medium containing 10 μM DEHP or MEHP for > 6 months. Treated and control cells were incubated with complete medium for 16 h, and cultured with or without anticancer drugs (10 μM) for 48 h. For the P-glycoprotein inhibition assay, control and treated cells were incubated with complete medium for 16 h, and cultured with or without tariquidar (10 μM) for 24 h. Whole cell lysates were collected and total protein concentration was determined using a dye-binding method based on the Bradford assay with a spectrophotometer (U-2800A; Hitachi, Tokyo, Japan). Proteins were separated in a 10–12% polyacrylamide gel and transferred onto a polyvinylidene difluoride (PVDF) membrane. Membranes were incubated with antibodies for drug-resistance and apoptosis markers (Supplementary Table 1) [10]. EMT and stemness markers were also detected in control and treated cells. The relative intensities of blots were quantified using ImageJ software. Numbers indicate densitometric analysis of protein expression levels normalized to corresponding control levels (L1) and α-tubulin (the last row).

### P-glycoprotein activity assay

A fluorometric MDR assay kit (Abcam) was used to measure P-glycoprotein activity. HCT116, HCT116 DEHP, and HCT116 MEHP cells (1.0 × 10^4^ cells/well) were seeded into 96-well flat, clear-bottom, black-wall microplates and incubated for 24 h. Cells were treated with the P-glycoprotein inhibitor, tariquidar (0, 0.1, 1, 10 or 100 μM) for 24 h. Next, 100 µl MDR dye-loading solution was added to each well and incubated at 37°C for 1 h in the dark. MDR indicator dye fluorescence was detected using a spectrophotometer (U-2800A; Hitachi) at excitation and emission wavelengths of 490 nm and 525 nm, respectively. All experiments were performed in triplicate.

### Cell proliferation analysis

Untreated, DEHP-, or MEHP-treated HCT116 or SW480 cells (10,000 cells/mL) were seeded overnight. Cells were then cultured in medium containing vehicle, DEHP (10 μM), or MEHP (10 μM), with or without tariquidar (10 μM) and irinotecan (10 μM) for 48 h. WST-1 reagent (100 μL, BioVision, USA) was added and incubated for 4 h at 37°C, and the absorbance at 450 nm was measured. All experiments were performed in triplicate.

### Cell migration assays

The Costar Transwell System (8-Transwellize polycarbonate membrane, 6.5-mm diameter; Corning, Inc., Corning, NY) was used to evaluate cell migration. HCT116, HCT116 DEHP, HCT116 MEHP, or HCT116 TGFβ cells (5 × 10^4^ cells in 100 μL serum-free medium) were added to the upper well, and 500 μL complete medium was added to the lower chamber. After 48 h incubation at 37°C and 5% CO_2_, cells on the top of the membrane were removed using a damp cotton swab, and cells that had migrated to the lower surface were fixed in methanol for 15 min at room temperature and stained with 1% crystal violet. Migration was quantified by counting migrated cells on the lower surface of the membrane in at least seven fields per chamber using a 10× objective. The wound-healing assay was performed using the Culture-Insert 2 Well system in µ-Dish 35 mm, with a defined 500-μm cell-free gap (Ibidi, Munich, Germany). Cells were seeded on multi-well plates and cultured with the insert. The insert was removed when cells reached 100% confluence. Cell migration rate was quantified by comparing images of the gap area at 0 and 24 h. All experiments were performed in triplicate.

### Sphere formation analysis

HCT116, HCT116 DEHP, and HCT116 MEHP cells (1 × 10^3^) were cultured in serum-free medium supplemented with B27, 100X insulin-transferrin-selenium solution (ITS-G; Gibco Life Technologies), 1% penicillin-streptomycin (Hyclone), 20 ng/mL EGF, and 10 ng/mL βFGF. After 2 weeks, the total number of spheres and sphere sizes in each well were assessed under a microscope. Sphere images were captured and measured using ImageJ.

### Statistical analysis

Clinical data are expressed as means ± standard deviation (SD). The Mann-Whitney *U* test was applied for between-group comparisons, and Student’s *t*-test was used to analyze differences between two treatment groups using GraphPad Prism 5 software. *P* < 0.05 was considered statistically significant. All assays were repeated at least three times independently.

## SUPPLEMENTARY MATERIALS FIGURES AND TABLES



## References

[1] Siegel R, Naishadham D, Jemal A (2013). Cancer statistics, 2013. CA Cancer J Clin.

[2] Chang HC, Horng JT, Lin WC, Lai HW, Chang CW, Chen TA (2012). Evaluation of the appropriate age range of colorectal cancer screening based on the changing epidemiology in the past 20 years in taiwan. ISRN Gastroenterol.

[3] Bae SU, Han YD, Cho MS, Hur H, Min BS, Baik SH, Lee KY, Kim NK (2016). Oncologic Outcomes of Colon Cancer Patients with Extraregional Lymph Node Metastasis: Comparison of Isolated Paraaortic Lymph Node Metastasis with Resectable Liver Metastasis. Ann Surg Oncol.

[4] Bockelman C, Engelmann BE, Kaprio T, Hansen TF, Glimelius B (2015). Risk of recurrence in patients with colon cancer stage II and III: a systematic review and meta-analysis of recent literature. Acta Oncol.

[5] Kazem MA, Khan AU, Selvasekar CR (2016). Validation of nomogram for disease free survival for colon cancer in UK population: A prospective cohort study. Int J Surg.

[6] Primrose JN (2010). Surgery for colorectal liver metastases. Br J Cancer.

[7] Anderson EC, Hessman C, Levin TG, Monroe MM, Wong MH (2011). The role of colorectal cancer stem cells in metastatic disease and therapeutic response. Cancers (Basel).

[8] Goldstein LJ, Galski H, Fojo A, Willingham M, Lai SL, Gazdar A, Pirker R, Green A, Crist W, Brodeur GM, Lieber M, Cossman J, Gottesman MM, Pastan I (1989). Expression of a multidrug resistance gene in human cancers. J Natl Cancer Inst.

[9] Tsuruo T, Naito M, Tomida A, Fujita N, Mashima T, Sakamoto H, Haga N (2003). Molecular targeting therapy of cancer: drug resistance, apoptosis and survival signal. Cancer Sci.

[10] Lee YK, Lin TH, Chang CF, Lo YL (2013). Galectin-3 silencing inhibits epirubicin-induced ATP binding cassette transporters and activates the mitochondrial apoptosis pathway via beta-catenin/GSK-3beta modulation in colorectal carcinoma. PLoS One.

[11] Chourasia MK, Jain SK (2003). Pharmaceutical approaches to colon targeted drug delivery systems. J Pharm Pharm Sci.

[12] Demoré B, Vigneron J, Perrin A, Hoffman MA, Hoffman M (2002). Leaching of diethylhexyl phthalate from polyvinyl chloride bags into intravenous etoposide solution. J Clin Pharm Therap.

[13] Anderson SE, Meade BJ (2014). Potential health effects associated with dermal exposure to occupational chemicals. Environ Health Insights.

[14] Tickner JA, Schettler T, Guidotti T, McCally M, Rossi M (2001). Health risks posed by use of Di-2-ethylhexyl phthalate (DEHP) in PVC medical devices: a critical review. Am J Ind Med.

[15] Erythropel HC, Maric M, Nicell JA, Leask RL, Yargeau V (2014). Leaching of the plasticizer di(2-ethylhexyl)phthalate (DEHP) from plastic containers and the question of human exposure. Appl Microbiol Biotechnol.

[16] Takeshita A, Inagaki K, Igarashi-Migitaka J, Ozawa Y, Koibuchi N (2006). The endocrine disrupting chemical, diethylhexyl phthalate, activates MDR1 gene expression in human colon cancer LS174T cells. J Endocrinol.

[17] Angelini A, Centurione L, Sancilio S, Castellani ML, Conti P, Di Ilio C, Porreca E, Cuccurullo F, Di Pietro R (2011). The effect of the plasticizer diethylhexyl phthalate on transport activity and expression of P-glycoprotein in parental and doxo-resistant human sarcoma cell lines. J Biol Regul Homeost Agents.

[18] National Toxicology Program (1997). Effect of Dietary Restriction on Toxicology and Carcinogenesis Studies in F344/N Rats and B6C3F1 Mice. Natl Toxicol Program Tech Rep Ser.

[19] National Toxicology Program (1997). NTP Toxicology and Carcinogenesis Studies of Butyl Benzyl Phthalate (CAS No. 85-68-7) in F344/N Rats (Feed Studies). Natl Toxicol Program Tech Rep Ser.

[20] National Toxicology Program (1995). NTP Toxicology and Carcinogenesis Studies of Diethylphthalate (CAS No. 84-66-2) in F344/N Rats and B6C3F1 Mice (Dermal Studies) with Dermal Initiation/ Promotion Study of Diethylphthalate and Dimethylphthalate (CAS No. 131-11-3) in Male Swiss (CD-1(R)) Mice. Natl Toxicol Program Tech Rep Ser.

[21] Ito Y, Yamanoshita O, Asaeda N, Tagawa Y, Lee CH, Aoyama T, Ichihara G, Furuhashi K, Kamijima M, Gonzalez FJ, Nakajima T (2007). Di(2-ethylhexyl)phthalate induces hepatic tumorigenesis through a peroxisome proliferator-activated receptor alpha-independent pathway. J Occup Health.

[22] Lin CH, Wu CY, Kou HS, Chen CY, Huang MC, Hu HM, Wu MC, Lu CY, Wu DC, Wu MT, Kuo FC (2013). Effect of Di(2-ethylhexyl)phthalate on Helicobacter pylori-Induced Apoptosis in AGS Cells. Gastroenterol Res Pract.

[23] Latini G, De Felice C, Presta G, Del Vecchio A, Paris I, Ruggieri F, Mazzeo P (2003). In utero exposure to di-(2-ethylhexyl)phthalate and duration of human pregnancy. Environ Health Perspect.

[24] Hsieh TH, Tsai CF, Hsu CY, Kuo PL, Lee JN, Chai CY, Wang SC, Tsai EM (2012). Phthalates induce proliferation and invasiveness of estrogen receptor-negative breast cancer through the AhR/HDAC6/c-Myc signaling pathway. FASEB J.

[25] Pocar P, Fiandanese N, Berrini A, Secchi C, Borromeo V (2017). Maternal exposure to di(2-ethylhexyl)phthalate (DEHP) promotes the transgenerational inheritance of adult-onset reproductive dysfunctions through the female germline in mice. Toxicol Appl Pharmacol.

[26] Chen HP, Pan MH, Chou YY, Sung C, Lee KH, Leung CM, Hsu PC (2017). Effects of di(2-ethylhexyl)phthalate exposure on 1,2-dimethyhydrazine-induced colon tumor promotion in rats. Food Chem Toxicol.

[27] Weidner LD, Fung KL, Kannan P, Moen JK, Kumar JS, Mulder J, Innis RB, Gottesman MM, Hall MD (2016). Tariquidar Is an Inhibitor and Not a Substrate of Human and Mouse P-glycoprotein. Drug Metab Dispos.

[28] Kavlock R, Boekelheide K, Chapin R, Cunningham M, Faustman E, Foster P, Golub M, Henderson R, Hinberg I, Little R, Seed J, Shea K, Tabacova S (2002). NTP Center for the Evaluation of Risks to Human Reproduction: phthalates expert panel report on the reproductive and developmental toxicity of di(2-ethylhexyl) phthalate. Reprod Toxicol.

[29] Lioy PJ, Hauser R, Gennings C, Koch HM, Mirkes PE, Schwetz BA, Kortenkamp A (2015). Assessment of phthalates/phthalate alternatives in children's toys and childcare articles: Review of the report including conclusions and recommendation of the Chronic Hazard Advisory Panel of the Consumer Product Safety Commission. J Expo Sci Environ Epidemiol.

[30] Green R, Hauser R, Calafat AM, Weuve J, Schettler T, Ringer S, Huttner K, Hu H (2005). Use of di(2-ethylhexyl) phthalate-containing medical products and urinary levels of mono(2-ethylhexyl) phthalate in neonatal intensive care unit infants. Environ Health Perspect.

[31] Silva MJ, Samandar E, Preau JL, Needham LL, Calafat AM (2006). Urinary oxidative metabolites of di(2-ethylhexyl) phthalate in humans. Toxicology.

[32] Doull J, Cattley R, Elcombe C, Lake BG, Swenberg J, Wilkinson C, Williams G, van Gemert M (1999). A cancer risk assessment of di(2-ethylhexyl)phthalate: application of the new U.S. EPA Risk Assessment Guidelines. Regul Toxicol Pharmacol.

[33] Buchta C, Bittner C, Heinzl H, Hocker P, Macher M, Mayerhofer M, Schmid R, Seger C, Dettke M (2005). Transfusion-related exposure to the plasticizer di(2-ethylhexyl)phthalate in patients receiving plateletpheresis concentrates. Transfusion.

[34] Yao PL, Lin YC, Richburg JH (2012). Mono-(2-ethylhexyl) phthalate (MEHP) promotes invasion and migration of human testicular embryonal carcinoma cells. Biol Reprod.

[35] Chen ML, Chen JS, Tang CL, Mao IF (2008). The internal exposure of Taiwanese to phthalate--an evidence of intensive use of plastic materials. Environ Int.

[36] Pallis M, Russell N (2000). P-glycoprotein plays a drug-efflux-independent role in augmenting cell survival in acute myeloblastic leukemia and is associated with modulation of a sphingomyelin-ceramide apoptotic pathway. Blood.

[37] Xu Y, Xia F, Ma L, Shan J, Shen J, Yang Z, Liu J, Cui Y, Bian X, Bie P, Qian C (2011). MicroRNA-122 sensitizes HCC cancer cells to adriamycin and vincristine through modulating expression of MDR and inducing cell cycle arrest. Cancer Lett.

[38] Yamada T, Takaoka AS, Naishiro Y, Hayashi R, Maruyama K, Maesawa C, Ochiai A, Hirohashi S (2000). Transactivation of the multidrug resistance 1 gene by T-cell factor 4/beta-catenin complex in early colorectal carcinogenesis. Cancer Res.

[39] Lee WK, Chakraborty PK, Thevenod F (2013). Pituitary homeobox 2 (PITX2) protects renal cancer cell lines against doxorubicin toxicity by transcriptional activation of the multidrug transporter ABCB1. Int J Cancer.

[40] Seo SB, Hur JG, Kim MJ, Lee JW, Kim HB, Bae JH, Kim DW, Kang CD, Kim SH (2010). TRAIL sensitize MDR cells to MDR-related drugs by down-regulation of P-glycoprotein through inhibition of DNA-PKcs/Akt/GSK-3beta pathway and activation of caspases. Mol Cancer.

[41] Chen FP, Chien MH (2014). Lower concentrations of phthalates induce proliferation in human breast cancer cells. Climacteric.

[42] Yurdakok Dikmen B, Alpay M, Kismali G, Filazi A, Kuzukiran O, Sireli UT (2015). *In Vitro* Effects of Phthalate Mixtures on Colorectal Adenocarcinoma Cell Lines. J Environ Pathol Toxicol Oncol.

[43] Cheng JX, Liu BL, Zhang X (2009). How powerful is CD133 as a cancer stem cell marker in brain tumors?. Cancer Treat Rev.

[44] Wright MH, Calcagno AM, Salcido CD, Carlson MD, Ambudkar SV, Varticovski L (2008). Brca1 breast tumors contain distinct CD44+/CD24- and CD133+ cells with cancer stem cell characteristics. Breast Cancer Res.

[45] Ma S, Chan KW, Lee TK, Tang KH, Wo JY, Zheng BJ, Guan XY (2008). Aldehyde dehydrogenase discriminates the CD133 liver cancer stem cell populations. Mol Cancer Res.

[46] O'Brien CA, Pollett A, Gallinger S, Dick JE (2007). A human colon cancer cell capable of initiating tumour growth in immunodeficient mice. Nature.

[47] Neuzil J, Stantic M, Zobalova R, Chladova J, Wang X, Prochazka L, Dong L, Andera L, Ralph SJ (2007). Tumour-initiating cells vs. cancer 'stem' cells and CD133: what's in the name?. Biochem Biophys Res Commun.

[48] Florek M, Haase M, Marzesco AM, Freund D, Ehninger G, Huttner WB, Corbeil D (2005). Prominin-1/CD133, a neural and hematopoietic stem cell marker, is expressed in adult human differentiated cells and certain types of kidney cancer. Cell Tissue Res.

[49] Park EK, Lee JC, Park JW, Bang SY, Yi SA, Kim BK, Park JH, Kwon SH, You JS, Nam SW, Cho EJ, Han JW (2015). Transcriptional repression of cancer stem cell marker CD133 by tumor suppressor p53. Cell Death Dis.

[50] Wei Y, Jiang Y, Zou F, Liu Y, Wang S, Xu N, Xu W, Cui C, Xing Y, Liu Y, Cao B, Liu C, Wu G (2013). Activation of PI3K/Akt pathway by CD133-p85 interaction promotes tumorigenic capacity of glioma stem cells. Proc Natl Acad Sci U S A.

[51] Haraguchi N, Ohkuma M, Sakashita H, Matsuzaki S, Tanaka F, Mimori K, Kamohara Y, Inoue H, Mori M (2008). CD133+CD44+ population efficiently enriches colon cancer initiating cells. Ann Surg Oncol.

[52] Ieta K, Tanaka F, Haraguchi N, Kita Y, Sakashita H, Mimori K, Matsumoto T, Inoue H, Kuwano H, Mori M (2008). Biological and genetic characteristics of tumor-initiating cells in colon cancer. Ann Surg Oncol.

[53] Petrelli F, Coinu A, Ghilardi M, Cabiddu M, Zaniboni A, Barni S (2015). Efficacy of oxaliplatin-based chemotherapy + bevacizumab as first-line treatment for advanced colorectal cancer: a systematic review and pooled analysis of published trials. Am J Clin Oncol.

[54] Fiteni F, Nguyen T, Vernerey D, Paillard MJ, Kim S, Demarchi M, Fein F, Borg C, Bonnetain F, Pivot X (2014). Cisplatin/gemcitabine or oxaliplatin/gemcitabine in the treatment of advanced biliary tract cancer: a systematic review. Cancer Med.

[55] Cortinovis D, Bidoli P, Zilembo N, Fusi A, Bajetta E (2008). Oxaliplatin doublets in non-small cell lung cancer: a literature review. Lung Cancer.

[56] Alcindor T, Beauger N (2011). Oxaliplatin: a review in the era of molecularly targeted therapy. Curr Oncol.

[57] Yang XQ, Li CY, Xu MF, Zhao H, Wang D (2015). Comparison of first-line chemotherapy based on irinotecan or other drugs to treat non-small cell lung cancer in stage IIIB/IV: a systematic review and meta-analysis. BMC Cancer.

[58] Shimazaki J, Motohashi G, Nishida K, Tabuchi T, Ubukata H, Tabuchi T (2014). Complete response of lung metastases from rectal cancer to combination first-line therapy of S-1 and irinotecan plus bevacizumab: A case report and review of the literature. Oncol Lett.

[59] Oostendorp LJ, Stalmeier PF, Pasker-de Jong PC, Van der Graaf WT, Ottevanger PB (2010). Systematic review of benefits and risks of second-line irinotecan monotherapy for advanced colorectal cancer. Anticancer Drugs.

[60] Chen MC, Lee NH, Hsu HH, Ho TJ, Tu CC, Chen RJ, Lin YM, Viswanadha VP, Kuo WW, Huang CY (2016). Inhibition of NF-kappaB and metastasis in irinotecan (CPT-11)-resistant LoVo colon cancer cells by thymoquinone via JNK and p38. Environ Toxicol.

[61] Abdallah EA, Fanelli MF, Souza E Silva V, Machado Netto MC, Gasparini Junior JL, Araújo DV, Ocea LM, Buim ME, Tariki MS, Alves Vda S, Piana de Andrade V, Dettino AL, Abdon Lopes de Mello C, Chinen LT (2016). MRP1 expression in CTCs confers resistance to irinotecan-based treatment in metastatic colorectal cancer. Int J Cancer.

[62] To KK, Poon DC, Wei Y, Wang F, Lin G, Fu LW (2016). Data showing the circumvention of oxaliplatin resistance by vatalanib in colon cancer. Data Brief.

[63] Zhang X, Xu P, Ni W, Fan H, Xu J, Chen Y, Huang W, Lu S, Liang L, Liu J, Chen B, Shi W (2016). Downregulated DYRK2 expression is associated with poor prognosis and Oxaliplatin resistance in hepatocellular carcinoma. Pathol Res Pract.

[64] Liu Z, Qiu M, Tang QL, Liu M, Lang N, Bi F (2010). Establishment and biological characteristics of oxaliplatin-resistant human colon cancer cell lines. Chin J Cancer.

[65] Mohn C, Hacker HG, Hilger RA, Gutschow M, Jaehde U (2013). Defining the role of MRP-mediated efflux and glutathione in detoxification of oxaliplatin. Pharmazie.

[66] Shukla S, Ohnuma S, Ambudkar SV (2011). Improving cancer chemotherapy with modulators of ABC drug transporters. Curr Drug Targets.

[67] Hsieh TH, Tsai CF, Hsu CY, Kuo PL, Lee JN, Chai CY, Hou MF, Chang CC, Long CY, Ko YC, Tsai EM (2012). Phthalates stimulate the epithelial to mesenchymal transition through an HDAC6-dependent mechanism in human breast epithelial stem cells. Toxicol Sci.

[68] Zhu H, Zheng J, Xiao X, Zheng S, Dong K, Liu J, Wang Y (2010). Environmental endocrine disruptors promote invasion and metastasis of SK-N-SH human neuroblastoma cells. Oncol Rep.

[69] Tsai CF, Hsieh TH, Lee JN, Hsu CY, Wang YC, Kuo KK, Wu HL, Chiu CC, Tsai EM, Kuo PL (2015). Curcumin Suppresses Phthalate-Induced Metastasis and the Proportion of Cancer Stem Cell (CSC)-like Cells via the Inhibition of AhR/ERK/SK1 Signaling in Hepatocellular Carcinoma. J Agric Food Chem.

[70] Wang YC, Tsai CF, Chuang HL, Chang YC, Chen HS, Lee JN, Tsai EM (2016). Benzyl butyl phthalate promotes breast cancer stem cell expansion via SPHK1/S1P/S1PR3 signaling. Oncotarget.

