# Supplementary material for: Phthalate exposure promotes chemotherapeutic drug resistance in colon cancer cells
Source: Oncotarget. 2017 Dec 8;9(17):13167–80. doi: 10.18632/oncotarget.23481 (PMC5862569; doi:10.18632/oncotarget.23481)
Supplement: Supplementary file 1 [file oncotarget-09-13167-s001.pdf]

# Phthalate exposure promotes chemotherapeutic drug resistance in colon cancer cells

## SUPPLEMENTARY MATERIALS

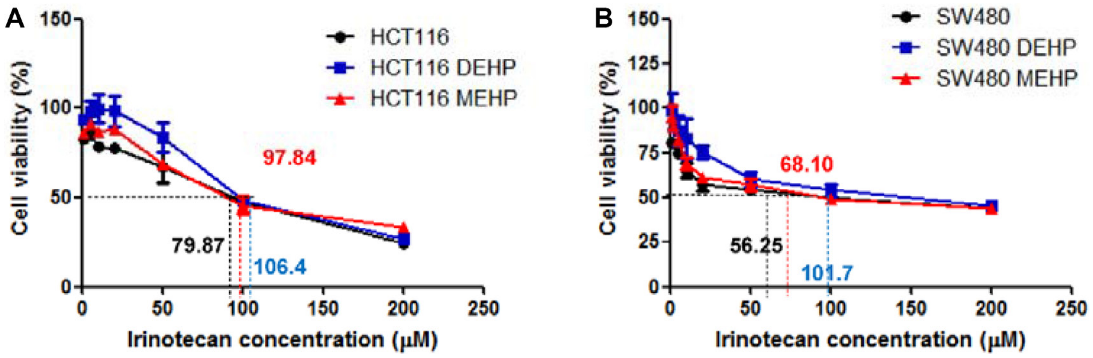


**Supplementary Figure 1:** The $IC_{50}$ values of irinotecan for Untreated, DEHP and MEHP treated (**A**) HCT116 or (**B**) SW480 cells. Untreated, DEHP and MEHP treated HCT116 or SW480 cells were plated in triplicates into 96-well plates at a density of 10,000 cells/ml. After 24 h, complete culture medium was changed into fresh serum-containing medium (10% FBS) containing DMSO (control) or tariquidar and indicated doses of irinotecan (Selleckchem). Cell viability 48 h after treatment was determined by WST-1 assay (BioVision, USA) according to manufacturer's instructions. Results are expressed as percentages of control, which was assigned 100% viability, and represented as the mean ± standard deviation (SD) of the triplicate wells. The $IC_{50}$ values of irinotecan for Untreated, DEHP and MEHP treated HCT116 are 80, 98, and 106 µM, respectively. The $IC_{50}$ values of irinotecan for Untreated, DEHP and MEHP treated SW480 are 56, 68, and 102 µM, respectively. The data are presented as the means ± standard deviation (SD) from at least three independent experiments.

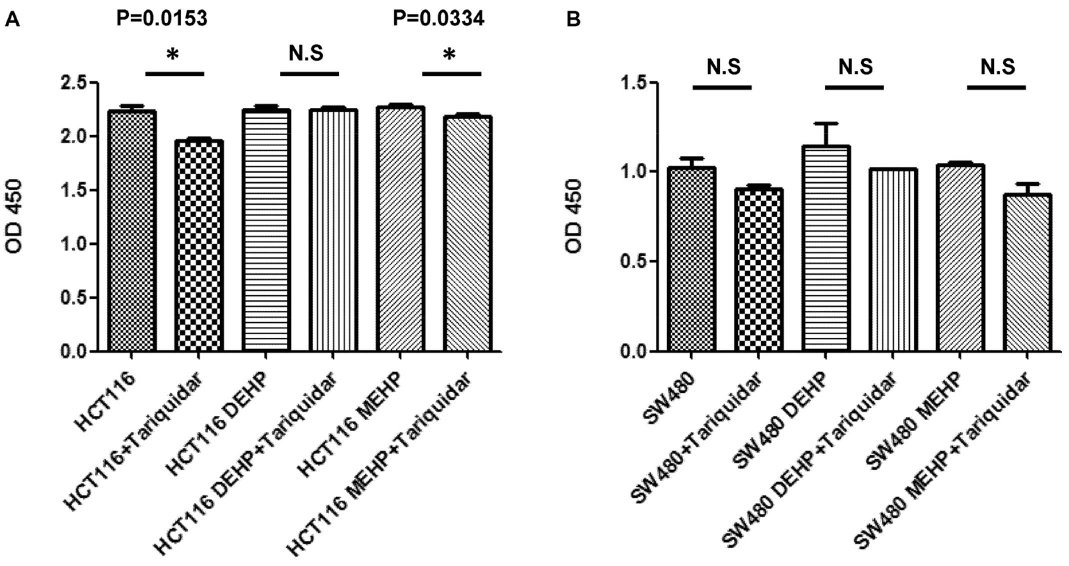


**Supplementary Figure 2:** Tariquidar showed low toxicity to (**A**) HCT116 or (**B**) SW480 cells. HCT116 and DEHP/ MEHP treated cells or SW480 cells and the DEHP/ MEHP treated cells were plated into 96-well plates at a density of 10,000 cells/ml. After 24 h, complete culture medium was changed into fresh serum-containing medium (10% FBS) containing DMSO (control) or tariquidar (10 μM). Cell proliferation was determined by WST-1 assay (BioVision, USA) according to manufacturer's instructions after 48 h followed by measuring the absorbance at 450 nm. The data are presented as the means ± standard deviation (SD) from at least three independent experiments. $^{*}P < 0.05$.

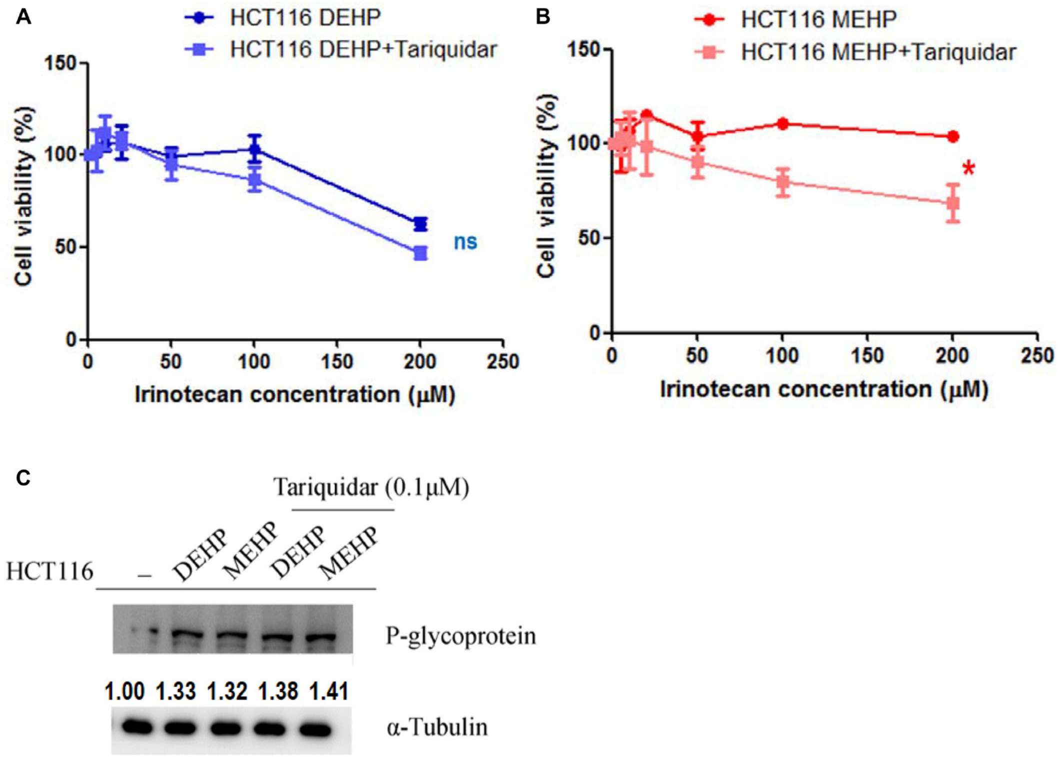


**Supplementary Figure 3: Tariquidar treatment reduced DEHP/MEHP-induced drug resistance by blocking drug efflux in HCT116 cells.** DEHP- or MEHP-treated HCT116 cells were incubated with or without tariquidar (0.1 μM) for 24 h and then challenged with irinotecan (0, 1, 5, 10, 20, 50, 100, or 200 μM) for 48 h. Tariquidar pretreatment decreased viability in irinotecan-challenged, DEHP (**A**) or MEHP (**B**) treated HCT116 cells. P-glycoprotein was not affected following tariquidar treatment (**C**) Data are presented as means ± standard deviation (SD) from at least three independent experiments. Numbers indicate densitometric analysis of protein expression levels normalized to corresponding control levels and α-tubulin (the last row).

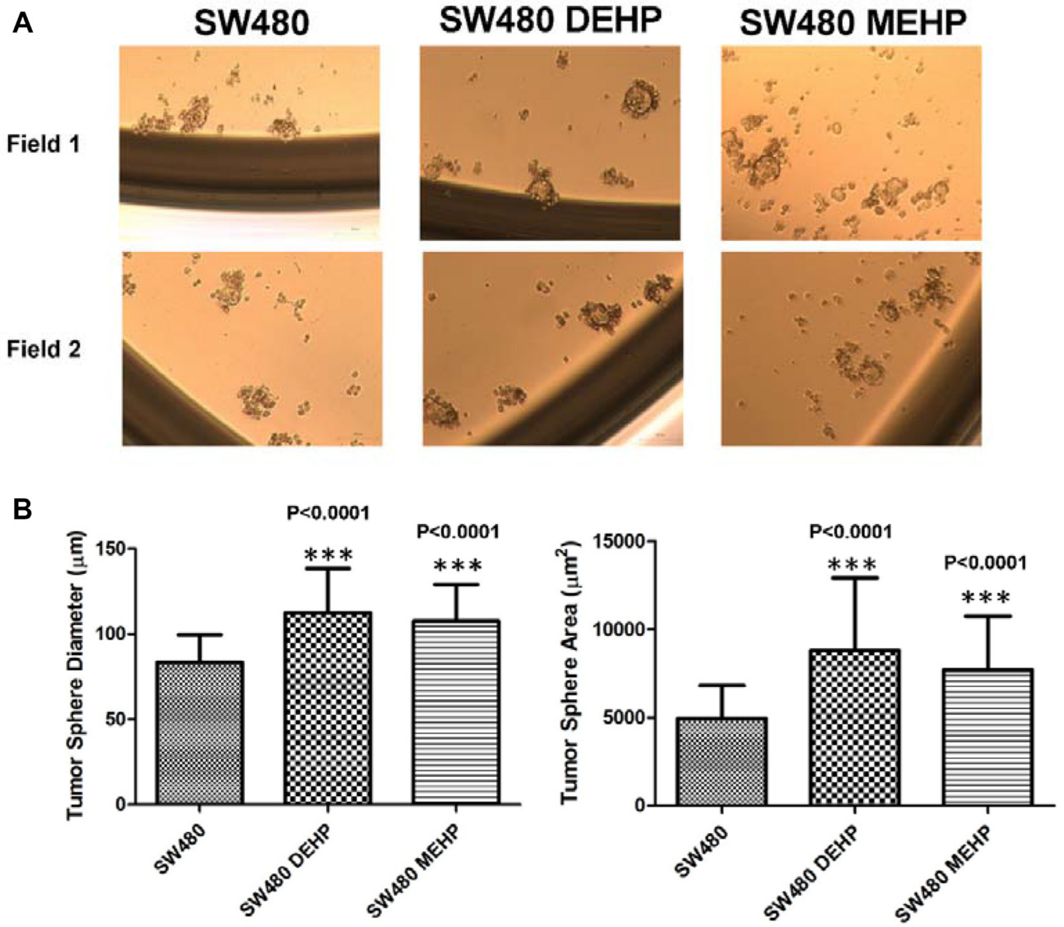


**Supplementary Figure 4: DEHP/MEHP/ treatment increases sphere formation in and DEHP/MEHP treated SW480 cells.** SW480 cells, SW480 DEHP and SW480 MEHP cells ($1 \times 10^3$) were cultured in serum-free medium in a low-attachment plate. After 2 weeks, the spheres sizes (diameter and area) in each well were counted under a microscope. The sphere images were captured (scale bar = 100 μm) and measured using ImageJ. The diameter and area of spheres were significantly increased in DEHP/MEHP treated SW480 cells. The data are presented as the means ± standard deviation (SD) from at least three independent experiments. $^{***}P < 0.001$, $^{###}P < 0.001$ compared with SW480 cells.

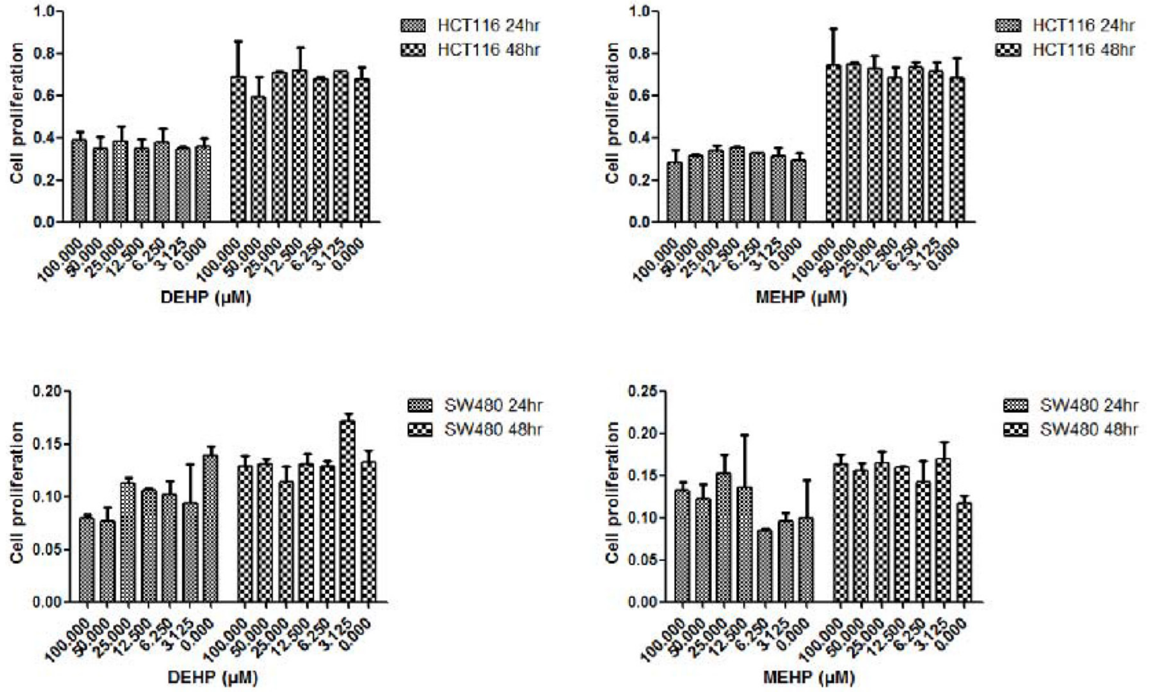


**Supplementary Figure 5: Cell viability of DEHP or MEHP treated HCT116 or SW480 cells.** HCT116 or SW480 cells were plated in triplicates into 96-well plates at a density of 10,000 cells/ml. After 24 h, complete culture medium was changed into fresh serum-containing medium (10% FBS) containing indicated doses of DEHP (100, 50, 25, 12.5, 6.25, 3.125 and 0 μM) or MEHP (100, 50, 25, 12.5, 6.25, 3.125 and 0 μM) and incubated for 24 and 48 h. Cell proliferation was determined by WST-1 assay (BioVision, USA) according to manufacturer's instructions. The data are presented as the means ± standard deviation (SD) from at least three independent experiments.

**Supplementary Table 1: List of antibodies used for western bloting**

| Item | Source |
|---|---|
| P-glycoprotein | GeneTex, San Antonio, TX |
| CD133 | Abnova Corporation, Walnut, CA |
| Bcl-2 | GeneTex, San Antonio, TX |
| Bax | GeneTex, San Antonio, TX |
| α-Tubulin | GeneTex, San Antonio, TX |
| MRP2 | Abcam, Cambridge, MA |
| pAKT | Abcam, Cambridge, MA |
| AKT | Abcam, Cambridge, MA |
| pERK | Abcam, Cambridge, MA |
| ERK | Abcam, Cambridge, MA |
| GSK-3α/β | Cell Signaling Technology, Danvers, MA |
| pGSK-3α/β (Ser21/9) | Cell Signaling Technology, Danvers, MA |
| β-catenin | GeneTex, San Antonio, TX |
| E-cadherin | Abcam, Cambridge, MA |
| N-cadherin | Abcam, Cambridge, MA |
| Vimentin | Abcam, Cambridge, MA |
| α SMA | Abcam, Cambridge, MA |
| Oct4 | GeneTex, San Antonio, TX |
| Sox2 | GeneTex, San Antonio, TX |
| Nanog | GeneTex, San Antonio, TX |
| Actin | Sigma-Aldrich, St. Louis, MO |
| HRP-conjugated goat anti-mouse IgG | Leadgene Biomedical,Taiwan |
| HRP-conjugated goat anti-rabbit IgG | Leadgene Biomedical,Taiwan |

**Supplementary Table 2: Gender and mean age of colon cancer patient among the four stages**

| | stage I | stage II | stage III | stage IV |
|---|---|---|---|---|
| Male | 19 | 25 | 25 | 23 |
| Female | 21 | 14 | 15 | 17 |
| Mean Age (SD) | 66.93 (11.99) | 62.26 (13.03) | 62.98 (10.75) | 60.80 (12.40) |
| Age Range | 37–85 | 39–88 | 45–81 | 31–85 |
